# Structural and Functional Studies on the Interaction of GspC and GspD in the Type II Secretion System

**DOI:** 10.1371/journal.ppat.1002228

**Published:** 2011-09-08

**Authors:** Konstantin V. Korotkov, Tanya L. Johnson, Michael G. Jobling, Jonathan Pruneda, Els Pardon, Annie Héroux, Stewart Turley, Jan Steyaert, Randall K. Holmes, Maria Sandkvist, Wim G. J. Hol

**Affiliations:** 1 Department of Biochemistry, Biomolecular Structure Center, University of Washington, Seattle, Washington, United States of America; 2 Department of Microbiology and Immunology, University of Michigan Medical School, Ann Arbor, Michigan, United States of America; 3 Department of Microbiology, University of Colorado School of Medicine, Aurora, Colorado, United States of America; 4 Department of Molecular and Cellular Interactions, VIB, Brussels, Belgium; 5 Structural Biology Brussels, Vrije Universiteit Brussel, Brussels, Belgium; 6 National Synchrotron Light Source, Brookhaven National Laboratory, Upton, New York, United States of America; Osaka University, Japan

## Abstract

Type II secretion systems (T2SSs) are critical for secretion of many proteins from Gram-negative bacteria. In the T2SS, the outer membrane secretin GspD forms a multimeric pore for translocation of secreted proteins. GspD and the inner membrane protein GspC interact with each other via periplasmic domains. Three different crystal structures of the homology region domain of GspC (GspC^HR^) in complex with either two or three domains of the N-terminal region of GspD from enterotoxigenic *Escherichia coli* show that GspC^HR^ adopts an all-β topology. N-terminal β-strands of GspC and the N0 domain of GspD are major components of the interface between these inner and outer membrane proteins from the T2SS. The biological relevance of the observed GspC–GspD interface is shown by analysis of variant proteins in two-hybrid studies and by the effect of mutations in homologous genes on extracellular secretion and subcellular distribution of GspC in *Vibrio cholerae.* Substitutions of interface residues of GspD have a dramatic effect on the focal distribution of GspC in *V. cholerae*. These studies indicate that the GspC^HR^–GspD^N0^ interactions observed in the crystal structure are essential for T2SS function. Possible implications of our structures for the stoichiometry of the T2SS and exoprotein secretion are discussed.

## Author Summary

Many bacterial pathogens affecting humans, animals and plants export diverse proteins across the cell membranes into the medium surrounding the bacteria. Some of these secreted proteins are involved in pathogenesis. One example is cholera toxin secreted by the bacterium *Vibrio cholerae*, a causative agent of cholera. The sophisticated type II secretion system is responsible for moving this toxin, and several other proteins, across the outer membrane. Here, we studied the interaction between the outer membrane pore of the type II secretion system, the secretin GspD, and the inner membrane protein GspC. We have solved three crystal structures of complexes between the interacting domains and identified critical contacts in the GspC–GspD interface. We also showed the importance of these contacts for assembly of the secretion system and for secretion of proteins by *V. cholerae*. Our studies provide a major piece in the puzzle of how the type II secretion system is assembled and how it functions. One day this knowledge might allow us to design compounds which interfere with this secretion process. Such compounds would be useful in the battle against bacteria affecting human health.

## Introduction

Many Gram-negative bacteria use a multi-protein type II secretion system (T2SS) to secrete a wide variety of exoproteins from the periplasm into the extra-cellular milieu [1], [2], [3], [4]. In *Vibrio cholerae* and enterotoxigenic *Escherichia coli* (ETEC), cholera toxin and the closely related heat-labile enterotoxin, in addition to other virulence factors, are secreted in their folded state across the outer membrane by the T2SS [5], [6], [7]. The T2SSs are composed of 12 to 15 different proteins that form three distinct subassemblies: (i) the inner membrane platform consisting of multiple copies each of GspC, GspF, GspL and GspM with an associated cytoplasmic secretion ATPase; (ii) the pseudopilus, a filamentous arrangement of multiple copies of five different pseudopilins; and (iii) a large, pore-forming outer membrane complex, mainly consisting of the secretin GspD [8], [9].

Secretins are multimeric outer membrane proteins composed of 50–70 kDa subunits and are among the largest outer membrane proteins known. The secretin superfamily has representatives in several other multi-protein complexes engaged in transport of large macromolecular substrates across the outer membrane [10] including the T2SS, the filamentous phage extrusion machinery [11], the type IV pilus system (T4PS) [12], [13], [14], and the type III secretion system (T3SS) [15], [16]. Of these systems, the T2SS is most closely related to the T4PS which assembles and disassembles long filamentous fibers on bacterial surfaces and is responsible for diverse functions including attachment to host cells, biofilm formation, DNA uptake and twitching motility [17], [18].

**Figure 1:**
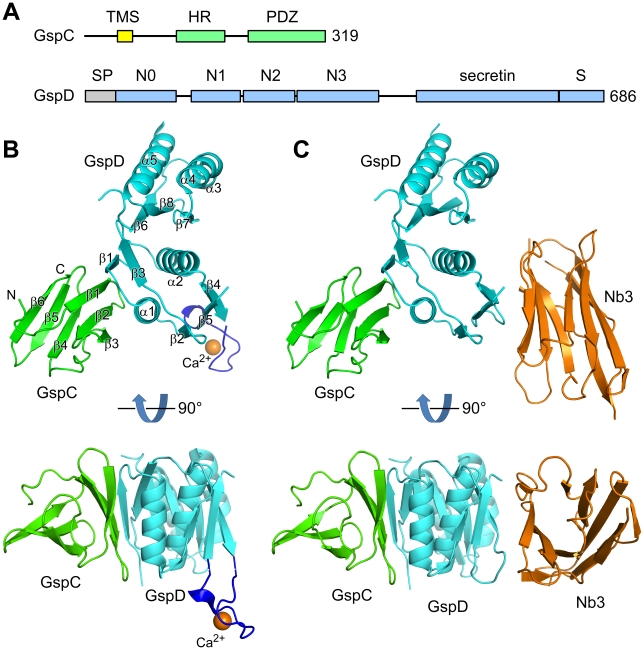
Structures of complexes of ETEC GspC^HR^ and GspD domains. (**A**) Schematic diagrams of the domain structures of GspC and GspD. TMS – transmembrane segment; SP – signal peptide. (**B**) Structure of the GspC^HR^–GspD^N0-N1^ binary complex. GspC, GspD and the LBT loop are colored green, cyan and blue, respectively. A Ca^2+^ ion that occupies the LBT metal binding site is shown as an orange sphere. Secondary structure elements are labeled. (**C**) Structure of the GspC^HR^–GspD^N0-N1-N2^–Nb3 ternary complex. The structure is shown in the same orientation as in (**B**) for the GspD domains. The orientation of the GspC^HR^ domain with respect to the GspD^N0^ domain is very similar in (**B**) and (**C**). Nanobody Nb3 is colored in orange. The N2 subdomain of GspD is statistically disordered in the crystal lattice (Figure S2). The structure of the GspC^HR^–GspD^N0-N1^–Nb3 ternary complex is essentially the same, despite the differences in GspD constructs, crystallization conditions and crystal forms (Figure S3).

The T2SS secretin GspD forms a dodecameric assembly according to electron microscopy studies [19], [20]. The C-terminal 300 to 400 residues of GspD contain the most conserved segments of the secretin superfamily, which form the actual outer membrane pore [21], [22], [23]. The N-terminal part of GspD consists of four domains: N0-N1-N2-N3 (Figure 1A) [19], [24]. The crystal structure of the N0-N1-N2 domains of the ETEC secretin GspD has been solved previously with the assistance of a single-domain llama antibody fragment or nanobody [24]. Nanobodies are the antigen-binding fragments (VHH) of heavy-chain-only camelid antibodies, which have been proven as effective crystallization chaperones for challenging targets, e.g. the T2SS pseudopilins complex [25], a trypanosomal editosome protein [26], and activated G-protein coupled receptor [27]. In the case of the secretin GspD^N0-N1-N2^ structure, nanobody Nb7 provided new crystal contacts and stabilized the N0-N1 domains lobe with respect to the N2 domain. The N0 domain is structurally related to domains from several proteins in bacterial multi-protein membrane complexes [28], [29], [30], [31], and to a domain of protein gp27 from T4-related bacteriophages [32]. As expected from sequence homology, the repeat N1 and N2 domains have the same fold, whereas the N3 domain is predicted to have a similar structure [24]. The fold of the N1 domain is different from that of the N0 domain and is structurally related to the eukaryotic type I KH (hnRNP K homology) domain [33]. By combining crystallographic and cryo-electron microscopy studies, it has been proposed that the N0, N1, N2 and N3 domains form the large periplasmic vestibule of the GspD dodecamer [20]. According to a number of biochemical studies, the outer membrane protein GspD has also been reported to interact with exoproteins [20], [34].

The inner membrane protein GspC consists of several domains: a short N-terminal cytoplasmic domain that is followed by the single transmembrane helix, a Pro-rich linker, the so-called homology region (HR) domain in the periplasm, a second linker and a C-terminal domain (Figure 1A) [35]. Most frequently, this C-terminal domain is a PDZ domain, but in some cases it is a coiled-coil domain [36], [37]. Crystal structures of the GspC PDZ domain showed that this domain can adopt open and closed conformations [38].

It has been shown *in vivo* in *V. cholerae* that GspC and GspD interact [39]. The interaction between GspC and GspD appears critical for the function, and possibly even for the assembly, of the T2SS [39]. Besides providing a physical link between the two membranes, either or both of these proteins or their interaction could also be important for exoprotein recognition, pseudopilus formation and release of the exoprotein through the GspD pore. Biochemically, we showed that the HR domain of GspC is the key part of GspC that interacts with the periplasmic GspD^N0-N1-N2^ [38]. This interaction was confirmed and further investigated recently in the plant pathogen *Dickeya dadantii,* a species previously called *Erwinia chrysanthemi* [40]. The interaction between GspC and GspD of *Xanthomonas campestris* has also been observed *in vitro* [37].

We report three structures of GspC^HR^ in complex with N-terminal domains of GspD that provide a structural basis to understand the functional interplay between the inner membrane platform and the outer membrane secretin of the T2SS. The observed interface led to the design of experiments to probe the importance of specific amino acids by biochemical and *in vivo* studies. Altering interface residues disabled the interaction of GspC and GspD in a bacterial two-hybrid system. It also abrogated protease secretion and had a dramatic effect on the localization of GspC in the cell envelope in *V. cholerae*. Together these results show the physiological importance of the molecular interactions observed between the inner and the outer platform. In addition, the resultant structure of the HR domain of GspC means that the structures of essentially all globular domains of the major T2SS proteins are presently known. The structures of ETEC GspC^HR^ in complex with N-terminal domains of GspD reported here are the first to reveal critical interactions between the inner membrane platform and the outer membrane complex of the T2SS at the atomic level.

## Results

### Structures of Three GspC–GspD Complexes from ETEC

A complex of ETEC GspC^HR^ and GspD^N0-N1-N2^ could be obtained but yielded only poorly diffracting crystals. To improve the quality of these crystals, we screened the same set of GspD specific nanobodies that had been used previously to solve the structure of GspD^N0-N1-N2^ [24] as crystallization chaperones for the GspC^HR^–GspD^N0-N1-N2^ complex. Using nanobody Nb3, we obtained crystals of a ternary ETEC GspC^HR^–GspD^N0-N1-N2^–Nb3 complex, which diffracted initially only to ∼5.5 Å resolution. Nevertheless, a partial molecular replacement structure revealed that the HR domain of GspC interacts with the lobe formed by the N0-N1 domains of GspD. To better characterize this interaction we also crystallized smaller complexes of GspC^HR^–GspD^N0-N1^ with or without nanobodies. To assist in crystallographic phasing, we also engineered a lanthanide-binding tag (LBT) into the N0 domain of GspD^N0-N1^ [41]. The LBT to GspD^N0-N1^ facilitated crystal growth and the resultant crystals of the binary GspC^HR^–GspD^N0-N1^ complex diffracted to better than 2.7 Å resolution, with the LBT engaged in multiple crystal contacts (Figure S1). The structure of this binary GspC^HR^–GspD^N0-N1^ complex was solved by molecular replacement and refined with good crystallographic and stereochemical statistics (Table 1). In parallel, we also obtained crystals and solved the 4 Å resolution structure of a ternary GspC^HR^–GspD^N0-N1^–Nb3 complex, and also improved the diffraction of crystals of the GspC^HR^–GspD^N0-N1-N2^–Nb3 complex to ∼4 Å resolution (Table 1, Figure S2).

**Table 1:** Data collection and refinement statistics.

	GspC^HR^–GspD^N0-N1^	GspC^HR^–GspD^N0-N1-N2^–Nb3	GspC^HR^–GspD^N0-N1^–Nb3
*Data collection*			
Wavelength (Å)	0.97946	0.97973	0.97946
Space group	*P*2_1_2_1_2_1_	*P*6_1_22	*P*6_4_22
Unit cell dimensions			
*a*, *b*, *c* (Å)	45.50, 76.81, 85.77	142.20, 142.20, 188.09	156.57, 156.57, 71.67
α, β, γ (deg)	90, 90, 90	90, 90, 120	90, 90, 120
Resolution (Å)	42.88–2.63 (2.77–2.63)^a^	43.93–4.05 (4.27–4.05)	41.69–4.00 (4.22–4.00)
Completeness (%)	99.9 (99.9)	99.8 (99.7)	99.9 (100)
Redundancy	3.9 (3.9)	7.7 (7.8)	7.7 (7.6)
*R*_merge_ (%)	13.5 (84.3)	8.5 (88.2)	15.9 (94.6)
*I*/σ(*I)*	11.0 (2.1)	16.7 (2.8)	10.0 (2.3)
*Refinement*			
Resolution (Å)	42.88–2.63		
*R*_work_/*R*_free_ (%)	21.3/26.5		
No. of reflections	8942		
No. of atoms	1775		
*B* factor (Å^2^)	31.2		
R.m.s. deviations			
Bond lengths (Å)	0.013		
Bond angles (deg)	1.314		
*Ramachandran values* b			
Favoured (%)	98.2		
Allowed (%)	1.8		

a Values in parenthesis are for the highest resolution shell.

b Molprobity [73], http://molprobity.biochem.duke.edu/.

The three multiprotein structures obtained from different crystal forms allow a detailed description of the interactions between GspC and GpsD. In all three structures, the N0 domain of GspD interacts exclusively with the HR domain of GspC. In the 2.63 Å resolution binary complex, the LBT introduced into GspD^N0^ faces away from the interface with GspC (Figure 1B). In the two low-resolution ternary complex structures, the nanobody Nb3 binds the N0 domain of GspD, opposite to the binding site of the HR domain of GspC (Figure 1C and Figure S3). In all three structures the HR domain binds in very similar orientation to GspD, relative to its N0 domain. Hence, neither the LBT nor the nanobody appears to affect the binding mode of GspC to GspD. Because the structure of the binary GspC^HR^–GspD^N0-N1^ complex has the highest resolution, this structure will be used below to analyze the specific features of the GspC–GspD interaction.

### Structure of the HR Domain of GspC

**Figure 2:**
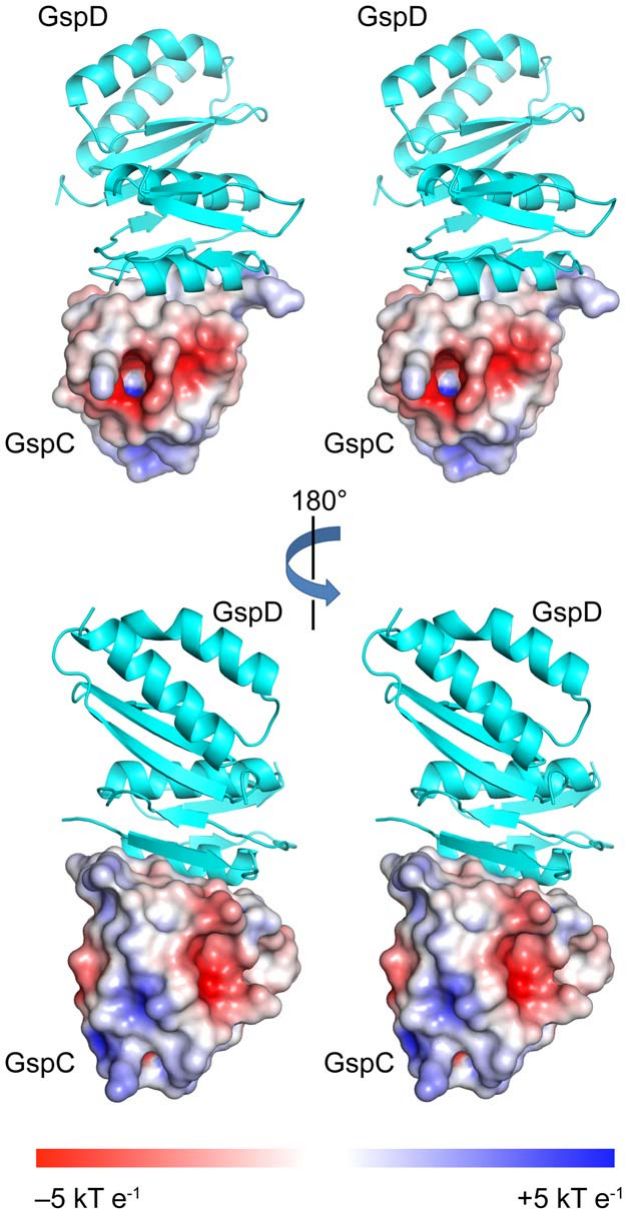
Surface characteristics of ETEC GspC^HR^. A stereo representation of surface charge distribution of GspC^HR^. The HR domain structure is from the binary complex with GspD^N0-N1^; the LBT is omitted for clarity. Note the deep pocket in the front surface of GspC^HR^ in the upper panel.

The HR domain folds into a β-sandwich formed by six consecutive β-strands arranged as two three-stranded anti-parallel β-sheets (Figure 1B). The residues between strands β3 and β4 adopt an approximately one-turn helical conformation. In its folded structure as seen in the complex with GspD^N0-N1^ (Figure 2), the distribution of charges on the surface of GspC^HR^ is quite uneven with the main hydrophobic surface interacting with GspD^N0^. Part of the remaining HR surface that is not involved in the GspD interaction (upper panel Figure 2) has a preponderance of negative charges and a deep pocket defined by residues Val127, Ile142 and Leu157. The other side of the HR domain (lower panel Figure 2) displays a mix of positive, negative and hydrophobic patches. The functions of these features during assembly and action of the T2SS, if any, remain to be determined.

### The Comparison of the HR Domain of GspC from the T2SS and PilP from the T4PS

**Figure 3:**
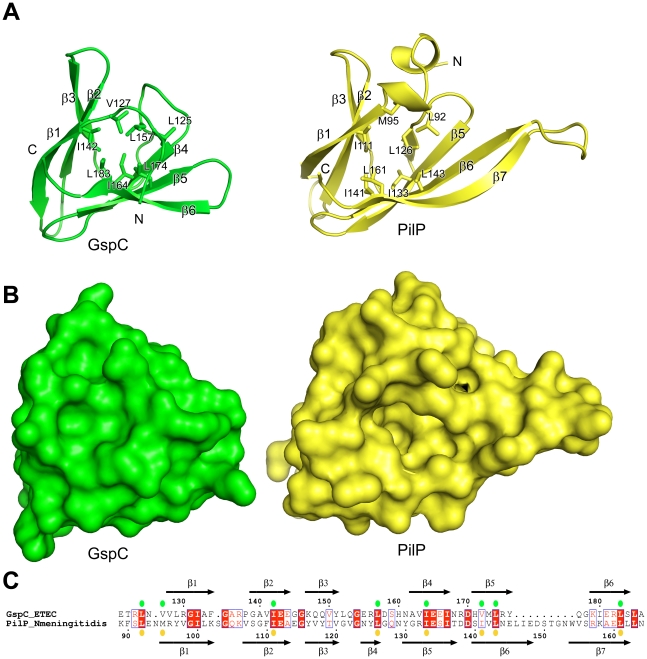
Comparison of GspC from the T2SS and PilP from the T4PS. (**A**) Structural superposition of the ETEC GspC^HR^ domain with the *N. meningitidis* PilP structure (PDB 2IVW) [42]. GspC and *Nm*PilP are colored in green and yellow, respectively. Flexible N- and C-terminal residues of *Nm*PilP are not shown for clarity. The conserved hydrophobic residues are shown as sticks. (**B**) Surface representation of GspC and PilP in the same orientation as in (**A**). The crevice on the surface of PilP is absent in GspC. (**C**) Sequence alignment of GspC and *Nm*PilP based on the structural superposition in (**A**). Secondary structure elements of GspC and *Nm*PilP are displayed above and below the sequences, respectively; the colored dots represent the conserved hydrophobic residues of GspC and *Nm*PilP.

The closest known structural homolog of the HR domain of ETEC GspC appears to be *Neisseria meningitidis* lipoprotein PilP (*Nm*PilP) which interacts with the secretin of the T4PS [42]. The HR domain of GspC and the core domain of *Nm*PilP superimpose with an r.m.s. deviation of 1.6 Å and 25% sequence identity over 59 residues (Figure 3). The structure of *Nm*PilP has been described as a β-sandwich composed of 7 β-strands [42]. Whereas residues 154–156 of GspC, corresponding to strand β4 of *Nm*PilP, make some main chain hydrogen bonds to residues in strand β4 of GspC (corresponding to β5 of *Nm*PilP), the secondary structure assignment algorithm of DSSP [43] does not classify these residues as β-structure.

A potential binding site has been described for the core *Nm*PilP domain [42]. It consists of a hydrophobic crevice on the open end of the β-sandwich. The residues that create this hydrophobic groove appear to be conserved between these two proteins from the T2SS and the T4PS when they are superimposed (Figure 3C). However, the area equivalent to the *Nm*PilP pocket is covered by residues N-terminal to strand β1 in ETEC GspC and, therefore, the *Nm*PilP pocket is absent in GspC (Figure 3B). These differences do not appear to stem from crystal contacts in the GspC^HR^–GspD^N0-N1^ structure. Moreover, these residues are well conserved (Figure S4A) and contribute to the hydrophobic core of the HR domain. The full implications of the global structural similarity between the core PilP domain of the T4PS and the HR domain of GspC from the T2SS remain to be established, but it is in line with several known similarities between the T2SS and T4PS [17], [18].

### The GspC–GspD Interface

**Figure 4:**
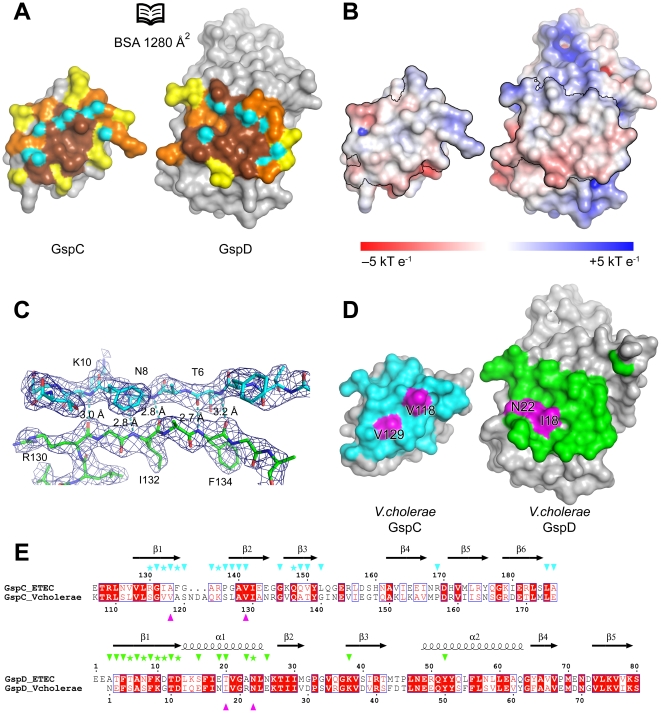
The interface of the GspC–GspD complex. (**A**) An ‘open book’ view of the GspC^HR^–GspD^N0-N1^ binary complex in surface representation. Residues in the interface are colored according to the degree of burial upon complex formation: yellow, up to 40% reduction in accessible surface area (ASA); orange, 40–70% reduction in ASA; and brown, more than 70% reduction in ASA. Atoms participating in intermolecular hydrogen bond formation are colored in cyan. (**B**) Same view as in (**A**) with the interaction surfaces colored according to the solvent accessible electrostatic potential. The interaction surface is contoured by black lines. (**C**) Anti-parallel β1^HR^–β1^N0^ interactions in the GspC^HR^–GspD^N0-N1^ complex. The upper strand is β1^N0^. Interacting residues are shown as sticks and labeled. Hydrogen bonds are shown as dashed lines. A σA-weighted 2*F*_O_–*F*_C_ electron density map contoured at 1.2 σ is shown as a dark blue mesh. (**D**) Interface surface of a homology model of the *V. cholerae* GspC–GspD complex [79]. Residues in the interface are colored according to the color of the interacting partner: GspD in cyan and GspC in green. The residues that were subjected to mutational analysis are colored in magenta and labeled. (**E**) Amino acid sequence alignments of the HR domains of GspC and the N0 domains of GspD from ETEC and *V. cholerae.* The corresponding secondary structure elements are shown above the sequences. Residues that make intermolecular Van der Waals contacts and H-bonds in the ETEC GspC^HR^–GspD^N0-N1^ complex are labeled by triangles and stars, respectively. The residues that were subjected to mutational analysis in *V. cholerae* GspC and GspD are indicated by magenta triangles underneath the alignments.

The interface between GspC^HR^ and GspD^N0^ buries 1280 Å^2^ of accessible surface area with a calculated *Δ*G of interaction of −5.4 kcal×mol^−1^ as assessed by the PISA server (Figure 4) [44]. The overall shape of the interface is relatively flat with a small concave area on the GspD surface. A total of 18 residues from GspC^HR^ and 19 residues from GspD^N0^ engage in a combination of hydrophobic interactions and hydrogen bonds. The first three β strands of GspC^HR^ and the first β strand plus the subsequent helix α1 of GspD^N0^ are the major contributors to the interface. The majority of the hydrogen bonds are formed by an antiparallel arrangement of strand β1 of GspC^HR^ and strand β1 of GspD^N0^ (Figure 4C). This β-strand augmentation is frequently observed in protein–protein interfaces [45]. Several nonpolar residues are engaged in intermolecular hydrophobic interactions, e.g. Ala133/Val141 from GspC, and Phe5/Phe9 from GspD. The hydrophobic nature of these interacting residues is well conserved, with GspC residue 133 being Ala, Leu, Val or Met; GspC residue 141 either a Val or Ile; GspD residue 5 a Phe or Tyr; and GspD residue 9 a Phe according to a family sequence alignment (Figure S4). Nonetheless, the GspC–GspD interface provides a species-specific connection point between outer and inner membrane assemblies of the T2SS as has been observed in genetic complementation studies [46], [47].

Based on the GspC^HR^–GspD^N0-N1^ structure, we selected several well-conserved interface residues for subsequent substitutional analysis. Ala133 and Val141 from ETEC GspC (equivalent to Val118 and Val129 from *V. cholerae* GspC, respectively; Figure 4E) and Thr20 from ETEC GspD (equivalent to Ile18 of *V. cholerae* GspD) are completely buried upon complex formation and are located in the center of the interacting surfaces (Figure 4D). Asn24 from ETEC GspD (equivalent to Asn22 of *V. cholerae* GspD) makes a hydrogen bond with the main chain oxygen of ETEC GspC Arg137. We evaluated the role of these residues on complex formation of truncated forms of GspC and GspD in a bacterial two-hybrid system and in a functional *V. cholerae* secretion assay *in vivo.* We also assessed the effect of interface substitutions on the distribution of GspC in the cell envelope of *V. cholerae*.

### Tests of GspC–GspD Interactions in the Bacterial Two-hybrid System

The effect of several interface substitutions on the ability of GspD to associate with GspC was assayed in a bacterial two-hybrid system based on reconstitution of activity of the catalytic domain of *Bordetella pertussis* adenylate cyclase when T18 and T25 fragments are fused to interacting proteins (see Methods) [48]. *Vc*GspD–T18 with a conservative Asn22Gln substitution retained the ability to interact with T25–*Vc*GspC and formed dark red colonies on indicator agar. In contrast, *Vc*GspD–T18 with either an Asn22Arg substitution or an Ile18Asp substitution lost the ability to interact with T25–*Vc*GspC and formed colorless colonies (Table 2). Two variants of T25–*Vc*GspC, with either Val118Arg or a Val129Arg substitution, also lost the ability to interact with *Vc*GspD–T18 and formed colorless colonies in the bacterial two-hybrid system.

**Table 2:** Characterization of GspC–GspD interaction in the bacterial two-hybrid system.

GspC	GspD	Interaction
wt^a^	wt	+
wt	Asn22Gln	+
wt	Asn22Arg	–
wt	Ile18Asp	–
Val118Arg	wt	–
Val129Arg	wt	–

a wt – wild type.

### Mutations in the GspC–GspD Interface Interfere with Protease Secretion in *V. cholerae*

**Figure 5:**
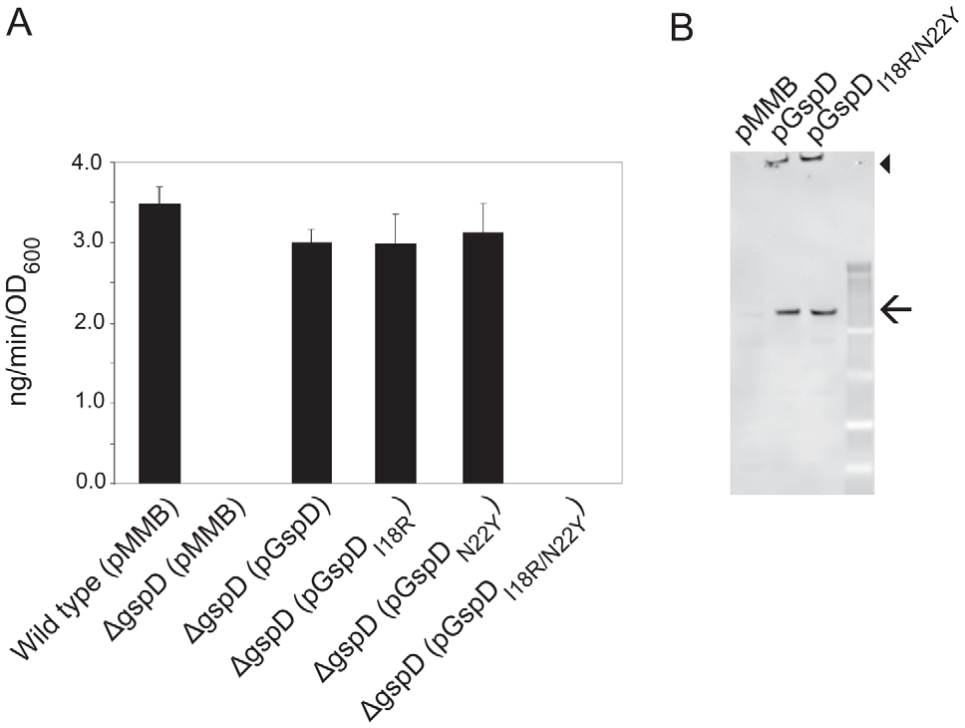
Simultaneous substitution of Ile18 and Asn22 in *V. cholerae* GspD results in inactivation of protease secretion by *V. cholerae.* (**A**) *V. cholerae* wild-type or *gspD* mutant strain (*Δ*gspD) containing either pMMB or pGspD variants were grown overnight in LB. Culture supernatants were separated from cells by centrifugation and tested for the presence of extracellular protease. The rate of hydrolysis was obtained from three independent experiments, and the results are presented with standard error. Both the pMMB vector control and pGspD_I18R/N22Y_ were below the limits of detection. (**B**) *V. cholerae gspD^–^* cells containing either pMMB or pGspD variants were disrupted and subjected to SDS-PAGE and immunoblotting with anti-GspD antibodies to determine the relative level of expression. The positions of molecular mass markers are shown. Arrow indicates monomeric GspD and arrowhead indicates multimeric GspD.

The functional importance of residues involved in the GspC–GspD interface was also assessed *in vivo* by monitoring the effect of the Ile18Arg and Asn22Tyr mutations in *Vc*GspD on the extracellular secretion of protease by *V. cholerae*. No protease secretion was observed when plasmid-encoded *Vc*GspD^Ile18Arg/Asn22Tyr^ was produced in a *V. cholerae* mutant strain lacking the gene encoding *Vc*GspD (Figure 5A), indicating that the simultaneous exchange of these two amino acids prevents protein secretion by the T2SS. The singly substituted variants, however, remained functional (Figure 5A). Immunoblot analysis of cell extracts from the *ΔgspD* mutant strain producing plasmid-encoded wild type and mutant *Vc*GspD showed that the double *Vc*GspD mutant protein was made at levels similar to that of wild-type *Vc*GspD (Figure 5B).

### Distribution of GFP-*Vc*GspC in *V. cholerae* Cells

**Figure 6:**
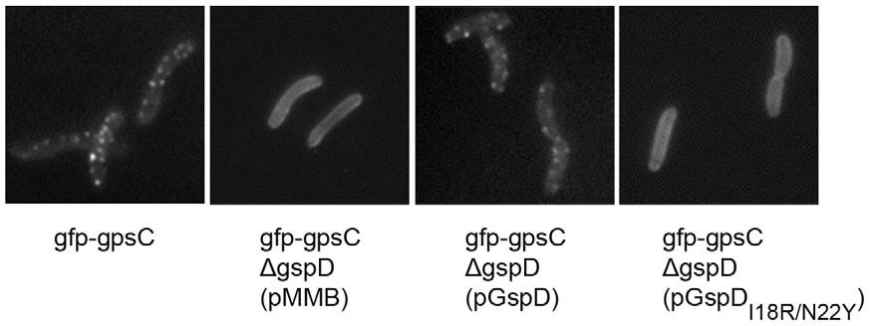
Differential localization in *V. cholerae* of GFP-GspC in the presence of GspD_I18R/N22Y_. Localization of chromosomally expressed GFP-GspC was examined in wild-type and *gspD* mutant backgrounds by fluorescence microscopy. GFP-GspC displayed a continuous membrane localization in the *gfp-gspC gspD^–^* strain (second panel) compared to the wild-type background (first panel). Punctate fluorescence was restored when the *gfp-gspC gspD^-^* strain was complemented with GspD on a plasmid (third panel). Expression of GspD_I18R/N22Y_ in the *gfp-gspC gspD^–^* strain resulted in membrane localization similar the pMMB vector control (fourth panel).

Using *V. cholerae* strains producing chromosomally encoded *Vc*GspC fused to the green fluorescent protein (GFP), we visually examined the effects of substitutions in the GspC–GspD interface on subcellular localization of GspC. GFP-*Vc*GspC forms fluorescent foci in the *V. cholerae* cell envelope, which disperse upon deletion of the gene encoding *Vc*GspD and reappear when the deletion strain is complemented with plasmid-encoded *Vc*GspD (Figure 6, first and second panels) [39]. The substitution of wild-type *Vc*GspD with *Vc*GspD^Ile18Arg/Asn22Tyr^ resulted in loss of fluorescent foci and dispersal of the fluorescence in a manner indistinguishable from cells that do not have the gene encoding *Vc*GspD at all (Figure 6, fourth panel). This result suggests that residues Ile18 and Asn22 of *Vc*GspD are critical for the incorporation of GFP-*Vc*GspC fusion protein into fluorescent foci, and supports the suggestion that the interaction between GspC and GspD observed in the crystal structure of GspC^HR^ in complex with GspD^N0-N1^ (Figure 4) is physiologically relevant. Based on these results, it appears that *Vc*GspD has to interact directly with *Vc*GspC in order to support its focal distribution in *V. cholerae*.

## Discussion

The current paper reveals for the first time key structural features of critical interactions between the outer membrane secretin GspD and the inner membrane protein GspC of the T2SS. The crystallographic studies benefited from the set of nanobodies against the N0-N1-N2 domains of GspD from ETEC [24] and from the incorporation of a lanthanide-binding tag (LBT) into ETEC GspD^N0^. The three GspC–GspD crystal structures elucidated reveal the same 1280 A^2^ interface involving the HR domain of GspC and the N0 domain of GspD. The crucial role of this interface was tested and confirmed by subsequent biochemical and functional studies. These results have interesting implications for our understanding of the T2SS and related secretion systems in many bacteria as discussed below.

### The Mutual Orientation of the N0 and N1 Domains of GspD

The structures of the first two domains of related secretins have been determined in two prior studies: ETEC GspD from the T2SS and EPEC EscC from the T3SS [24], [49]. The relative orientations of the N0 and N1 domains in these two studies appeared to be remarkably different: when the N1 domains of the T2SS and T3SS secretins are superimposed, the N0 domains are rotated by not less than 143 degrees [10]. This raises an important question as to the actual orientation of these two domains in the T2SS and T3SS secretins.

Regarding the T2SS, the relative orientations of the N0 and N1 GspD domains can now be compared in two high resolution structures, i.e. in the current structure of the binary complex of ETEC GspC^HR^ and GspD^N0-N1^, and in the previously determined binary complex of ETEC GspD^N0-N1-N2^ in complex with Nb7 [24]. The linker between the N0 and N1 subdomains is disordered in both these high resolution structures. The interface and relative orientation of the N0 and N1 subdomains, however, is essentially the same in the two structures despite the binding of either Nb7 or the presence of the LBT insertion into the N0 domain: the superposition of the two N0 domains results in an r.m.s. deviation of 0.49 Å for 72 Cα pairs (Figure S5). Taking also into account the two new low resolution structures of the ternary complexes of GspC^HR^–GspD^N0-N1^–Nb3 and GspC^HR^–GspD^N0-N1-N2^–Nb3 (Figure S3), then the N0-N1 lobe in the T2SS secretin GspD is observed as the same compact unit in four different crystal structures, independent of the presence or absence of a GspC^HR^ domain, Nb molecules or crystal contacts. The available data suggest that the N0-N1 orientation in GspD is a characteristic feature in the T2SS. However, we cannot exclude the possibility that the relative orientation of the N0 and N1 domains may change as the secretin oligomerizes. Only high resolution structures of the dodecameric secretin will resolve this question.

Since the N0-N1 lobe of the T2SS secretin fits well into the cryo-electron microscopy reconstruction of *Vc*GspD [20], and the N0 and N1 domains of the T3SS secretin fit well into a cryo-electron microscopy density of the *Salmonella typhimurium* needle complex [16], it might be that the N0 and N1 domains of these related secretins adopt different mutual orientations in the assembled T2SS and T3SS *in vivo* as observed in crystals. Obviously further studies are required to confirm this hypothesis where it also should be kept in mind that secretins are dynamic proteins and multiple orientations of N-terminal secretin domains might transiently occur during the secretion process [10].

### The GspC–GspD Crystal Structure and Functional Studies

The crystal structure indicates that a number of residues are critical for the interactions of ETEC GspC and GspD (Figure 4). Moreover, these residues are conserved in the family sequence alignment (Figure S4). As many mutants and other useful reagents have already been generated and developed for studies of the T2SS in *V. cholerae*, subsequent probing of the importance of these residues for the interaction was carried out in three different ways using *V. cholerae* GspC and GspD homologues. The two-hybrid studies showed that substitutions Val118Arg and Val129Arg in *Vc*GspC, and Asn22Arg in *Vc*GspD, abrogated the interaction between GspC^HR^ and GspD^N0-N1-N2^ from *V. cholerae* (Table 2). The secretion of protease by *V. cholerae* was also greatly diminished by substitutions Ile18Arg/Asn22Tyr in full length *Vc*GspD (Figure 5). Finally, the same Ile18Arg/Asn22Tyr variant of *Vc*GspD altered the distribution of full-length *Vc*GspC in the inner membrane of *V. cholerae*, possibly by interfering with normal assembly of the inner membrane platform of the T2SS (Figure 6). Taking all data together, we conclude that the substitutions altering the interface of GspC with GspD in *V. cholerae* affect the interactions of GspC with GspD as demonstrated both in a bacterial two-hybrid system and by analysis of protease secretion by the T2SS in *V. cholerae*.

Interactions between GspC and GspD from *D. dadantii* have been recently investigated [40]. This study confirmed the interactions between GspC^HR^ and the N-terminal domains of GspD reported earlier for *V. vulnificus* homologs [38]. A GST-fusion of residues 139–158 of *Dd*GspC (corresponding to residues 168–187 in ETEC GspC) co-purified with both *Dd*GspD^N0^ and *Dd*GspD^N1-N2-N3^ [40]. The 139–158 residues of *Dd*GspC were therefore designated as secretin interacting peptide (SIP). In a homology model of *Dd*GspC^HR^–GspD^N0-N1^ complex, based on our crystal structure, this fragment is located far from the interface (Figure S6). It appears that this segment forms an anti-parallel pair of β-strands, β5 and β6, in the ETEC GspC^HR^ crystal structure, with β6 at the surface and β5 located between strands β6 and β4 (Figure S6). Furthermore, the substitutions introduced into the *Dd*GspC 139–158 fragment had no effect on the interaction with *Dd*GspD^N0^, whereas one substitution, Val143Ser, prevented *Dd*GspC interaction with *Dd*GspD^N1-N2-N3^ [40]. The same substitution, when introduced into full length *Dd*GspC, also interfered with secretion in *D. dadantii*. We also mapped these substitutions onto the homology model of the *Dd*GspC–GspD complex and it is clear that none of them are buried in the GspC–GspD interface (Figure S6). The only substitution that had an effect on secretion, Val143Ser, replaces a buried hydrophobic residue in the core of *Dd*GspC^HR^ with a polar residue that would likely be detrimental to the HR domain stability. This is in agreement with the finding that this substitution in GST-*Dd*GspC_128-272_ resulted in a protein that is degraded in the cells [40]. A more conservative Val143Ala substitution in full length *Dd*GspC appeared to largely support secretion of pectinases, in agreement with the less drastic change of the nature of the side chain, which could result in a larger proportion of properly folded protein than in the case of the Val143Ser variant. Therefore, the ETEC GspC^HR^–GspD^N0-N1^ structure explains several experimental results of the studies on *Dd*GspC–GspD interactions [40]. The observations that a GST-fusion of the *Dd*GspC 139–158 fragment is capable of interacting with fragments of the secretin in the absence of both the rest of the HR domain and the rest of the secretin, and of interfering with pectinase secretion when over-expressed in wild type *D. dadantii*, are difficult to interpret precisely. Additional studies are required to show that such interactions are not the result of non-specific interactions, possibly due to exposed hydrophobic residues of the peptide which are normally buried in the complete HR domain.

### T2SS Stoichiometry

**Figure 7:**
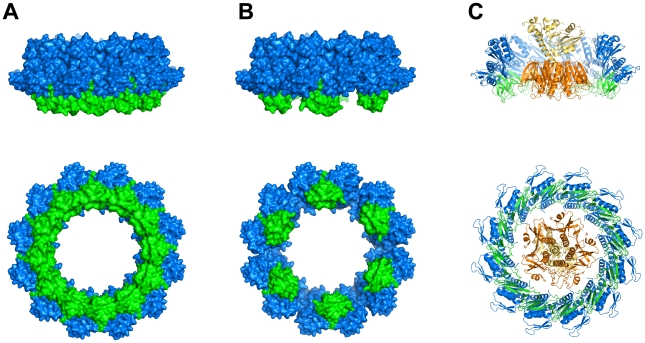
Combining structural data of GspD and GspC and the exoprotein cholera toxin. (**A**) Two perpendicular views of the dodecameric ring of GspD^N0-N1^ (blue) obtained from crystallographic and electron microscopy studies [20], [24] with a dodecameric ring of GspC^HR^ (green) assembled as described in the text. (**B**) Two perpendicular views of the same dodecameric ring of GspD^N0-N1^ (blue) shown in (**A**) with six GspC^HR^ subunits (green) binding to alternating GspD subunits as described in the text. (**C**) Two perpendicular views of the exoprotein cholera toxin (B pentamer in gold, A subunit in yellow) positioned inside the dodecameric GspC^HR^–GspD^N0-N1^ ring depicted in (**A**). The five-fold axis of the B-pentamer is aligned by hand with the twelve-fold axis of the ring. The orientation of the AB_5_ hexamer with respect to the dodecamer is otherwise arbitrary. The cross-section of the double dodecamer of GspD^N0-N1^ and GspC^HR^ is just sufficient to permit binding of the toxin heterohexamer. Obviously, the alternative assembly shown in (**B**) would also provide sufficient space for the toxin to bind at this location.

The implications of the GspC^HR^–GspD^N0^ interactions unraveled by our studies for the architecture of the T2SS are intriguing. The three new structures in the current paper show that one GspC^HR^ domain interacts with one GspD^N0^ domain, which suggests a 1∶1 ratio of GspC and GspD in the assembled T2SS. Since the stoichiometry of full length GspC and GspD has not been established yet in the context of a functional T2SS, it is of interest to see if the current complex of GspC^HR^–GspD^N0^ is compatible with the dodecameric ring of GspD^N0-N1^ derived recently by a combination of crystallographic and electron microscopy studies [20], [24]. Superimposing the GspC^HR^–GspD^N0^ complex twelve times onto the N0-domains of the GspD^N0-N1^ ring results in a double ring structure where the GspC^HR^ subunits added do not interfere with the formation of the GspD^N0-N1^ ring. Although this procedure does result in some clashes between the subunits of the GspC^HR^ ring, specifically between residues of the β2-β3 loop of one subunit and residues just prior to β6 in a neighboring subunit, small conformational changes in these loops, or minor adjustments in the mutual orientation of domains in the GspD^N0^ ring, or both, might alleviate these close contacts. If this would be the case, the GspD dodecamer would interact with twelve GspC subunits in the assembled T2SS (Figure 7A). Alternatively, only alternating GspD subunits of the dodecameric secretin might interact with GspC^HR^, obviously removing close contacts between the then well separated GspC^HR^ subunits. In this case, the GspD dodecamer would interact with six GspC subunits (Figure 7B).

These two alternatives for the interface of the outer membrane complex and the inner membrane platform can be combined with previous studies on the T2SS even though the ratio between GspC and the other components of the inner-membrane platform complex is currently unknown. Yet, the following observations are of interest for the T2SS stoichiometry puzzle:

(i) the secretion ATPase GspE of the T2SS has been suggested to be a hexamer [50], [51];

(ii) the cytoplasmic domain of the inner membrane T2SS protein GspL forms a 1∶1 complex with GspE [52];

(iii) homologs of GspM and of the cytoplasmic domain of GspL from the T4PS have been reported to form heterodimers [53], [54];

(iv) there are a few cases of gene fusion of the T4PS proteins PilP and PilO (e.g. *Pseudomonas putida* PilO-PilP, Uniprot entry Q88CU9) in the T4PS. PilP is a GspC^HR^ homolog (Figure 3) and PilO is proposed to be a homolog of the inner membrane protein GspM from the T2SS [54], [55]. The presence of PilO–PilP fusions may imply a 1∶1 stoichiometry of these proteins in the T4PS and, in view of the homology between the T4PS and the T2SS, a GspM:GspC ratio of 1∶1 in the T2SS as well.

These four observations suggest that GspE, GspL, GspM and GspC might be present in an equimolar ratio in the inner membrane platform. In view of the likely hexameric nature of GspE, this implies the presence of six subunits of each of these proteins in the assembled T2SS. If the GspD dodecamer would interact with six GspC subunits (Figure 7B), then this arrangement would agree well with six subunits each of GspC, GspL, GspM and GspE in the inner membrane platform. If a GspD dodecamer, however, would interact with twelve GspC subunits in the assembled T2SS (Figure 7A), then, a stoichiometry mismatch is likely to occur somewhere along the GspC–GspL–GspM–GspE chain of interactions in the inner membrane platform. This could be possible in spite of the evidence in points (i) to (iv) above for an equimolar ratio of these four proteins in the T2SS since e. g. points (iii) and (iv) are rather indirect and derived from observations on T4PS proteins. Clearly, the stoichiometry of the T2SS remains a fascinating topic for further studies, where the number of GspF subunits, the only T2SS protein which spans the inner membrane multiple times, also remains to be determined.

### Implications for Exoprotein Secretion

Another major outstanding question is the recognition of exoproteins by the T2SS. Interestingly, the inner diameter of the dodecameric GspC^HR^–GspD^N0-N1^ double ring is ∼68 Å, which implies that a large exoprotein like the cholera toxin AB_5_ heterohexamer [56] just fits into this ring (Figure 7C). This is in agreement with recent electron microscopy studies which indicate that the B-pentamer of cholera toxin can bind to the entrance of the GspD periplasmic vestibule [57]. The periplasmic domains of GspD and of GspC have been implicated in this crucial exoprotein recognition function [34], [46], [57], [58], [59], but the specific details of exoprotein–T2SS interactions remain to be uncovered. The accumulation of recent structural and biochemical data provides a platform for asking increasingly precise questions regarding the many remaining mysteries still pertaining to the architecture and mechanism of the sophisticated T2SS.

## Methods

### Expression and Purification of GspC and GspD for Crystallization

ETEC GspD^N0-N1-N2^ (residues 1–237; numbering corresponds to mature protein sequence) was expressed and purified as described [24]. The DNA sequence corresponding to residues 1–165 of ETEC GspD was PCR amplified and cloned into the pCDF-NT vector to obtain a GspD^N0-N1^ expression construct. pCDF-NT is a modified pCDF-Duet1 vector (Novagen) encoding an N-terminal His_6_-tag sequence and a TEV protease cleavage site. The DNA sequence corresponding to residues 122–186 of ETEC GspC was PCR amplified and cloned into a pCDF-NT vector to obtain a GspC^HR^ expression construct.

A lanthanide binding tag (LBT) was introduced into GspD^N0-N1^ construct in order to assist with crystallographic phase determination and promote crystal formation. In order to decrease the flexibility of the LBT, we introduced it into the loop between two adjacent β-strands rather than at the termini. The design was based on the crystal structure of ubiquitin with the double LBT (PDB 2OJR) [41] where two β-strands flank one of the LBT. The LBT sequence YIDTNNDGYIEGDEL was inserted between residues Met70 and Val74 of GspD^N0^ (Figure S4B) using the polymerase incomplete primer extension method [60]. While this manuscript was in preparation, a similar approach for the LBT insertion was successfully applied to a model protein, interleukin-1β [61].

GspD^N0-N1^ was expressed at 25°C in BL21(DE3) cells (Novagen) in LB medium containing 50 µg×ml^–1^ streptomycin. Protein production was induced with 0.5 mM IPTG. Cells were harvested 3 h after induction. GspD^N0-N1^ variants with or without LBT were purified by Ni-NTA agarose (Qiagen) chromatography followed by His_6_-tag cleavage with TEV protease; a second Ni-NTA chromatography to remove His_6_-tag, uncleaved protein and His-tagged TEV protease; and a final size-exclusion chromatography using Superdex 75 column (GE Healthcare). GspC^HR^ was expressed and purified under same conditions as GspD^N0-N1^. The proteins were concentrated, flash-frozen [62] and stored at −80°C. Se-Met-labeled proteins were expressed using metabolic inhibition of methionine biosynthesis [63] and purified using the protocols for native proteins.

### Purification of Nanobodies

The nine nanobodies generated against ETEC GspD^N0-N1-N2^ were expressed and purified as described previously [24].

### Crystallization, Data Collection and Structure Determination

ETEC GspC^HR^, GspD^N0-N1-N2^ and individual nanobodies were mixed at 1∶1∶1 molar ratio, concentrated to 4–8 mg×ml^−1^ total protein concentration and subjected to crystallization conditions screening by the vapor diffusion method at 4 or 21°C. The crystallization conditions were identified using SaltRx (Hampton Research) and JCSG+ (Qiagen) screens. The complex of GspC^HR^–GspD^N0-N1-N2^–Nb3 was crystallized in 1.2 M lithium sulfate, 0.1 M Tris-HCl pH 7 at 4°C. The crystals were gradually transferred to precipitant solution supplemented with 30% glycerol and flash-frozen in liquid nitrogen. Initial crystals diffracted to 5.5 Å resolution and optimized crystals with Se-Met substituted GspD^N0-N1-N2^ showed improved diffraction to 4.6 Å. Data were processed and scaled using XDS [64]. The structure was solved by molecular replacement using Phaser [65]; the search models included the GspD^N0-N1^ structure (PDB 3EZJ) [24], a camelid antibody fragment (PDB 1QD0) [66], and a homology model of GspC^HR^ obtained using the I-TASSER server [67] and the *N. meningitidis* PilP structure as template (PDB 2IVW) [42]. The N2 domain of GspD could not be located in the electron density maps due to statistical disorder (Figure S2).

The complex of GspC^HR^–GspD^N0-N1^ with an engineered LBT in the GspD^N0^ domain was crystallized in 0.9 M magnesium sulfate, 0.1 M bis-tris propane pH 7.0 at 21°C. The crystals were transferred to precipitant solution supplemented with 20% ethylene glycol and flash-frozen in liquid nitrogen. The structure of the GspC^HR^–GspD^N0-N1^ complex was solved by molecular replacement using Phaser and rebuilt using Buccaneer [68] and Coot [69]. The metal binding site of the LBT appears to be occupied by a Ca^2+^ ion based on the electron density and the B factor values after refinement (Figure S1). Most likely, Ca^2+^ ions were acquired during *E. coli* expression, which prevented Tb^3+^ ions binding during treatment of purified protein according to a published protocol [41]. The capture of ions during heterologous expression by Ca^2+^ binding proteins has been observed previously for the major pseudopilin GspG [70]. The structure was refined with REFMAC [71] using translation, libration and screw-rotation displacement (TLS) groups defined by the TLSMD server [72]. The quality of the structure was assessed using the Molprobity server [73].

The ternary GspC^HR^–GspD^N0-N1^–Nb3 complex was crystallized in 0.7 M sodium citrate, 0.1 M bis-tris propane, pH 7.0 at 21°C. The crystals were cryoprotected using 20% ethylene glycol. The structure of the GspC^HR^–GspD^N0-N1^–Nb3 complex was solved by molecular replacement using Phaser with refined GspC^HR^ and GspD^N0-N1^ fragments from our GspC^HR^–GspD^N0-N1^ structure as search models (Figure S3).

Protein–protein interfaces were evaluated using the PISA server [44]; structural homologs were searched for using the DALI server [74]; the electrostatic surface potential was calculated using APBS [75]; figures were prepared using PyMol [76].

### Two Hybrid Analysis of *Vc*GspC and *Vc*GspD Domain Interactions

Interaction between protein domains was detected by the ability of fusion proteins containing the enzymatically inactive T18 and T25 fragments of adenylate cyclase toxin from *Bordetella pertussis* to confer adenylate cyclase activity (and the ability to ferment maltose and form red colonies on maltose-MacConkey plates) to a *cyaA* mutant *E. coli* strain as described previously [48]. *E. coli* DC8F′ is a *cyaA*::Km^R^ derivative of the strain MM294 (*endA1 hsdR17 glnV44 thi-1*) with the Tc^R^ F′ *lacI^q^* Tn*10* from XL1blue (Stratagene). Plasmids pCT25*Vc*GspC (encoding a T25–*Vc*GspC fusion protein) and pA*Vc*GspDT18 (encoding a *Vc*GspD–T18 fusion protein) were separately transformed into *E. coli* DC8F′, and transformants were selected on LB-Cm or LB-Ap plates, respectively. Each of the resulting transformants formed white colonies when streaked onto maltose MacConkey plates and incubated at 30°C. In contrast, when both plasmids were transformed together into *E. coli* DC8F′, the resulting transformants formed red colonies when streaked onto maltose MacConkey plates, demonstrating a productive protein-protein interaction between the *Vc*GspC and *Vc*GspD domains of the T25–*Vc*GspC and *Vc*GspD–T18 fusion proteins, bringing together the T18 and T25 fragments to form active cyclase. A positive control also demonstrated a productive protein-protein interaction between CTA1_R7K_T18 and CT25ARF6 fusion proteins in *E. coli* DC8F′ and formation of red colonies on maltose MacConkey agar, as reported previously [48]. Negative controls failed to demonstrate any productive protein-protein interaction between the CTA1_R7K_T18 and T25*Vc*GspC fusion proteins or between the*Vc*GspDT18 and CT25ARF6 fusion proteins in *E. coli* DC8F′.

### Cloning of Sequences Encoding Soluble Cytoplasmic Domains of *Vc*GspC and *Vc*GspD into Two Hybrid Vectors

A DNA sequence encoding residues 53–305 of *Vc*GspC (AAA58784.1) was amplified by PCR using the primers EpsCXF and EpsCHIIIR adding *Xba*I (Leu-Glu frame) and a stop codon-*Hin*dIII sites at the 5′ and 3′ ends respectively. This product was cloned in frame after the T25 domain in place of ARF6 in pXCT25arf6 (pCT25ARF6 from [48] but with a vector *Xba*I site deleted) to generate pCT25*Vc*GspC. Similarly the coding sequence for residues 25–294 of *Vc*GspD (AAA58785.1) was amplified with primers EpsDNdeIF and EpsDClaR which add *Nde*I (and Met codon) and *Cla*I (Ser-Met frame) sites at the 5′ and 3′ ends respectively; this PCR product was cloned in place of the CTA1 gene in pCTA1_R7K_T18 [48] to generate pA*Vc*GspDT18. The primers sequence information is available upon request. Specific mutations in the *eps* gene domains (encoding GspC or GspD) in pA*Vc*GspDT18 and pCT25*Vc*GspC were generated by SOE-PCR [77] or by subcloning of a PCR fragment performed with a restriction site containing mutagenic primer and a vector primer, followed by recloning into the parental vector. All clones were verified in-frame and correct by DNA sequencing to ensure no additional PCR-generated mutations.

### Generation of *Vc*GspD Mutants

The *ΔgpsD* strain of *V. cholerae,* a *gfp-gspC ΔgspD* strain, and the complementing pMMB-*gspD* plasmid were constructed previously [39]. Mutations were introduced in the *gspD* gene of *V. cholerae* with the QuikChange II site-directed mutagenesis kit (Stratagene) using pBAD-gspD as a template. Primers used for the site change in *gspD*_I18R_ and *gspD*_N22Y_ were 5′-GAATTTATCAATCGTGTGGGACGCAATC-3′, 5′-GATTGCGTCCCACACGATTGATAAATTC-3′ and their reverse complements, respectively. *gspD*_I18R/N22Y_ was then constructed using pBAD-gspD_I18R_ as a template and the above primers specific for the *gspD*_N22Y_ site change. All mutations were verified by sequencing. Following sequencing, the *gspD* variants of *V. cholerae* obtained were cloned into the low-copy-vector pMMB67 using restriction enzymes *BamH*I and *Sph*I.

### Detection of Secreted Protease Activity

*V. cholerae* cultures were grown overnight at 37°C in Luria broth supplemented with 100 µg×ml^−1^ thymine, 200 µg×ml^−1^ carbenicillin, and 20 µM IPTG and centrifuged to separate the supernatant and cellular material. The supernatants were centrifuged once more, and the protease activity was measured as described previously [78].

### Microscopy

Cultures of *V. cholerae* were grown overnight at 37°C in M9 medium containing 0.4% casamino acids, 0.4% glucose, and 100 µg×ml^−1^ thymine; diluted 50–fold into fresh medium; and grown to mid-log phase before observation. Plasmids were maintained with 50 and 200 µg×ml^−1^ carbenicillin in log-phase and overnight cultures, respectively. Plasmid expression was induced with IPTG as described above. For fluorescence microscopy of live cells, cultures were mounted on 1.5% low-melting temperature agarose pads prepared with M9 glucose medium. All microscopy was performed with a Nikon Eclipse 90i fluorescence microscope equipped with a Nikon Plan Apo VC100 (1.4 numerical aperture) oil immersion objective and a Cool SNAP HQ2 digital camera. Captured images were analyzed with NIS-Elements imaging software (Nikon).

### Accession Numbers

Atomic coordinates and structure factors have been deposited in the Protein Data Bank (http://www.pdb.org) with accession code 3OSS.

## Supporting Information

Figure S1 The lanthanide binding tag (LBT) in the GspC^HR^–GspD^N0-N1^ crystal structure. (**A**) Stereoview of the LBT in the GspC^HR^–GspD^N0-N1^ crystal structure. The σA-weighted 2*F*_O_–*F*_C_ electron density map is displayed as a grey mesh at the 1 σ level. The Ca^2+^ ion is shown as an orange sphere; a coordinating water molecule as a red sphere. (**B**) The LBT makes several crystal contacts in the lattice.
(TIF)

Figure S2 Crystal structure of the GspC^HR^–GspD^N0-N1-N2^–Nb3 ternary complex. (**A**) SDS-PAGE analysis of crystals. Lane 1, molecular weight standards; lane 2, purified GspC^HR^; lane 3, purified GspD^N0-N1-N2^; lane 4, purified Nb3; lane 5, GspC^HR^–GspD^N0-N1-N2^–Nb3 complex before crystallization; lane 6, drop which did not yield crystals; lane 7, recovered crystal after washing in artificial mother liquor. The GspD^N0-N1-N2^ chain is intact after crystallization. (**B**) Molecular replacement structure of the GspC^HR^–GspD^N0-N1-N2^–Nb3 complex. GspD^N0-N1-N2^ and Nb3 are Se-Met substituted proteins. Se-Met residues are shown as sticks. The anomalous difference map at the 3.5 σ level is shown as a magenta mesh and clearly indicates selenium sites. (**C**) Crystal packing of GspC^HR^–GspD^N0-N1-N2^–Nb3 viewed along the crystallographic *c* axis of space group *P*6_1_22. GspC^HR^ is in green, GspD^N0-N1-N2^ in cyan, Nb3 in orange. The Cα atoms of the last residue in the N1 domain (A165) are shown as cyan spheres. The N2 domains are facing long channels in the crystal lattice and are statistically disordered since SDS-PAGE analysis of dissolved crystals shows the full length of the GspD^N0-N1-N2^ chain [see lane 7 in (**A**) above].
(TIF)

Figure S3 The GspC^HR^–GspD^N0^ interface in three crystal forms. The N0-N1 domains are colored cyan; the HR domains green and magenta; Nb3 nanobodies orange. (**A**) Two crystallographically related ternary GspC^HR^–GspD^N0-N1-N2^–Nb3 complexes in contact with each other in crystals with space group *P*6_1_22. (**B**) Two crystallographically related ternary GspC^HR^–GspD^N0-N1^–Nb3 complexes in contact in crystals with space group *P*6_4_22. Comparison with (**A**) above shows that the ternary complexes in these two crystal forms are very similar. The 2-fold crystallographic contacts are essentially the same in the two different crystal forms. (**C**) The GspC^HR^ chain has the same orientation with respect to GspD^N0^ in three different crystal forms. The superposition of the three GspC^HR^–GspD^N0^ complexes is based on GspD^N0-N1^ only. The HR domain of GspC^HR^–GspD^N0-N1^ is depicted in dark green; the HR domain of GspC^HR^–GspD^N0-N1-N2^–Nb3 in light green; and the HR domain of GspC^HR^–GspD^N0-N1^–Nb3 in magenta.
(TIF)

Figure S4 Sequence alignments of selected GspC proteins and GspD^N0^ domains. Residues that make intermolecular Van der Waals contacts and H-bonds in the ETEC GspC^HR^–GspD^N0-N1^ complex are labeled by triangles and stars, respectively. (**A**) Sequence alignment of GspC proteins. The secondary structure elements are shown at the top as determined from the ETEC GspC^HR^–GspD^N0-N1^ structure and the *V. cholerae* GspC^PDZ^ structure (PDB 2I4S) [38]. (**B**) Sequence alignment of GspD^N0^ domains. The secondary structure elements are shown at the top as determined from the ETEC GspC^HR^–GspD^N0-N1^ structure. The position and sequence of the LBT are shown.
(TIF)

Figure S5 The structure of GspD^N0-N1^ is virtually the same in the GspC^HR^–GspD^N0-N1^ and GspD^N0-N1-N2^–Nb7 structures. A stereoview of a superposition of GspD^N0-N1^ from the GspC^HR^–GspD^N0-N1^ complex (cyan) and the GspD^N0-N1-N2^–Nb7 complex (purple, PDB 3EZJ) [24]. The superposition is based on the N0 domain only (r.m.s.d. 0.49 Å for 72 Cα atoms). The mutual orientation of the N0 and N1 domains is very similar indeed.
(TIF)

Figure S6 Analysis of the *Dickeya dadantii* GspC^HR^–GspD^N0-N1^ complex. (**A**) A homology model of the *D. dadantii* (previously *Erwinia chrysanthemi*) GspC–GspD complex. The structures of the HR domain of *Dd*GspC (light green) and the N0-N1 domains of *Dd*GspD (light blue) were obtained by homology modeling based on our new structure of the ETEC GspC^HR^–GspD^N0-N1^ complex (grey) as template, using the SWISS-MODEL server (http://swissmodel.expasy.org/) [79]. (**B**) Mutations of *Dd*GspC. The residues in the interface of *Dd*GspC-GspD in the homology model are shown as sticks. A previously suggested interaction region SIP (secretin interacting peptide) that corresponds to residues 139–159 is highlighted in orange [40]. The residues which have been subjected to mutational analysis (R142, V143, V144, R150, E152 and Y157) are shown as sticks and labeled. The mutant *Dd*GspC proteins R142I, V144A, R150L, E152A and Y157A fully supported secretion of pectinases in *D. dadantii*. Note that, in contrast to the other residues, V143 is completely buried in the model and the substitution V143S leads to decreased secretion [40]. For further discussion see main text. (**C**) Sequence alignments of GspC^HR^ and GspD^N0^ from ETEC and *D. dadantii*. Secondary structure elements are shown above alignment according to ETEC GspC^HR^–GspD^N0-N1^ crystal structure. A previously suggested interaction region SIP that corresponds to residues 139–159 is indicated by an orange bar. The residues which have been subjected to mutational analysis (R142, V143, V144, R150, E152 and Y157) are highlighted by circles.
(TIF)
