# Supplementary figures and images for: Structural and Functional Studies on the Interaction of GspC and GspD in the Type II Secretion System
Source: PLoS Pathog. 2011 Sep 8;7(9):e1002228. doi: 10.1371/journal.ppat.1002228 (PMC3169554; doi:10.1371/journal.ppat.1002228)

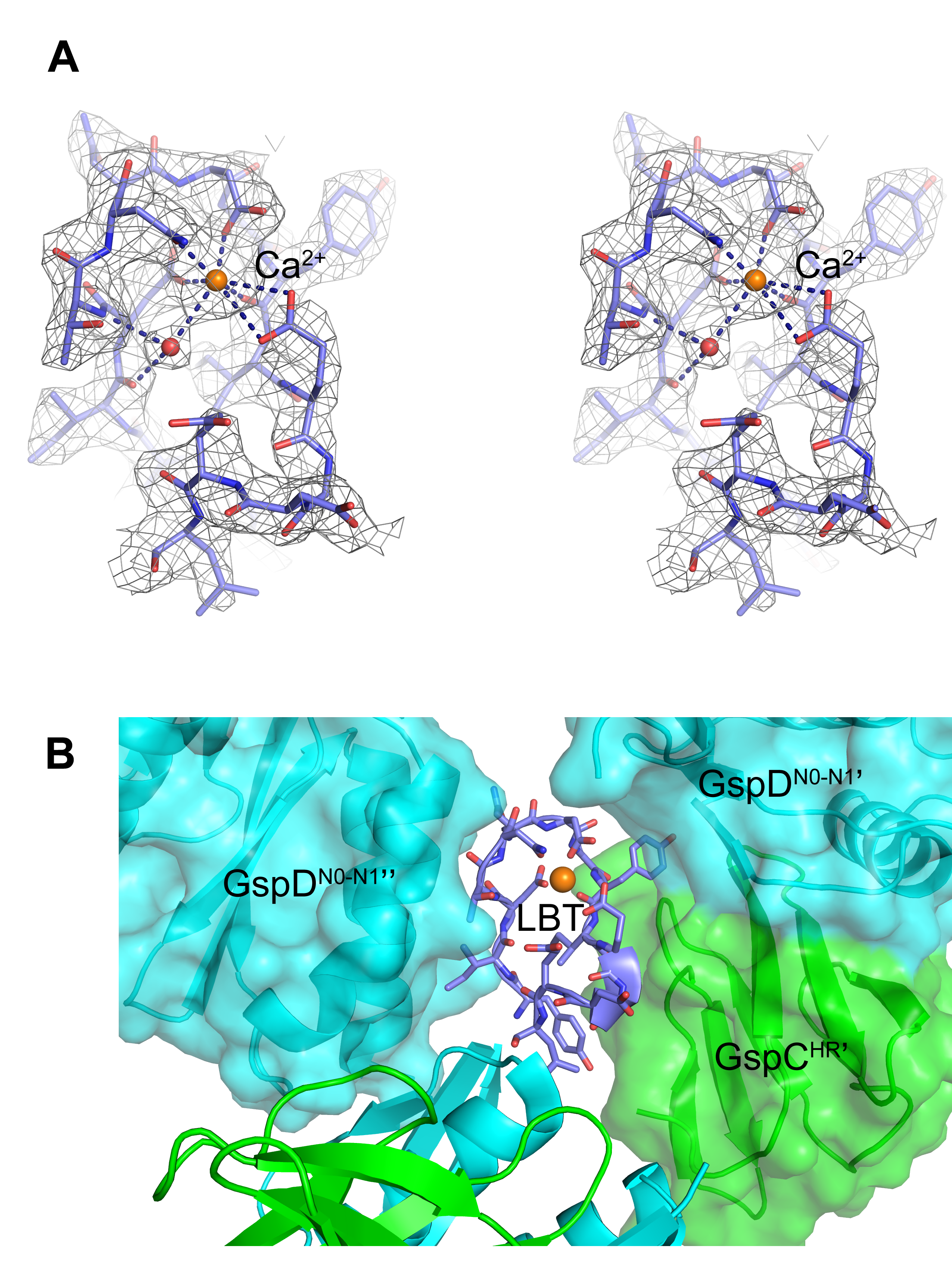

Supplement: Figure S1 — The lanthanide binding tag (LBT) in the GspCHR–GspDN0-N1 crystal structure. (A) Stereoview of the LBT in the GspCHR–GspDN0-N1 crystal structure. The σA-weighted 2F O–F C electron density map is displayed as a grey mesh at the 1 σ level. The Ca2+ ion is shown as an orange sphere; a coordinating water molecule as a red sphere. (B) The LBT makes several crystal contacts in the lattice. (TIF) [file ppat.1002228.s001.tif]

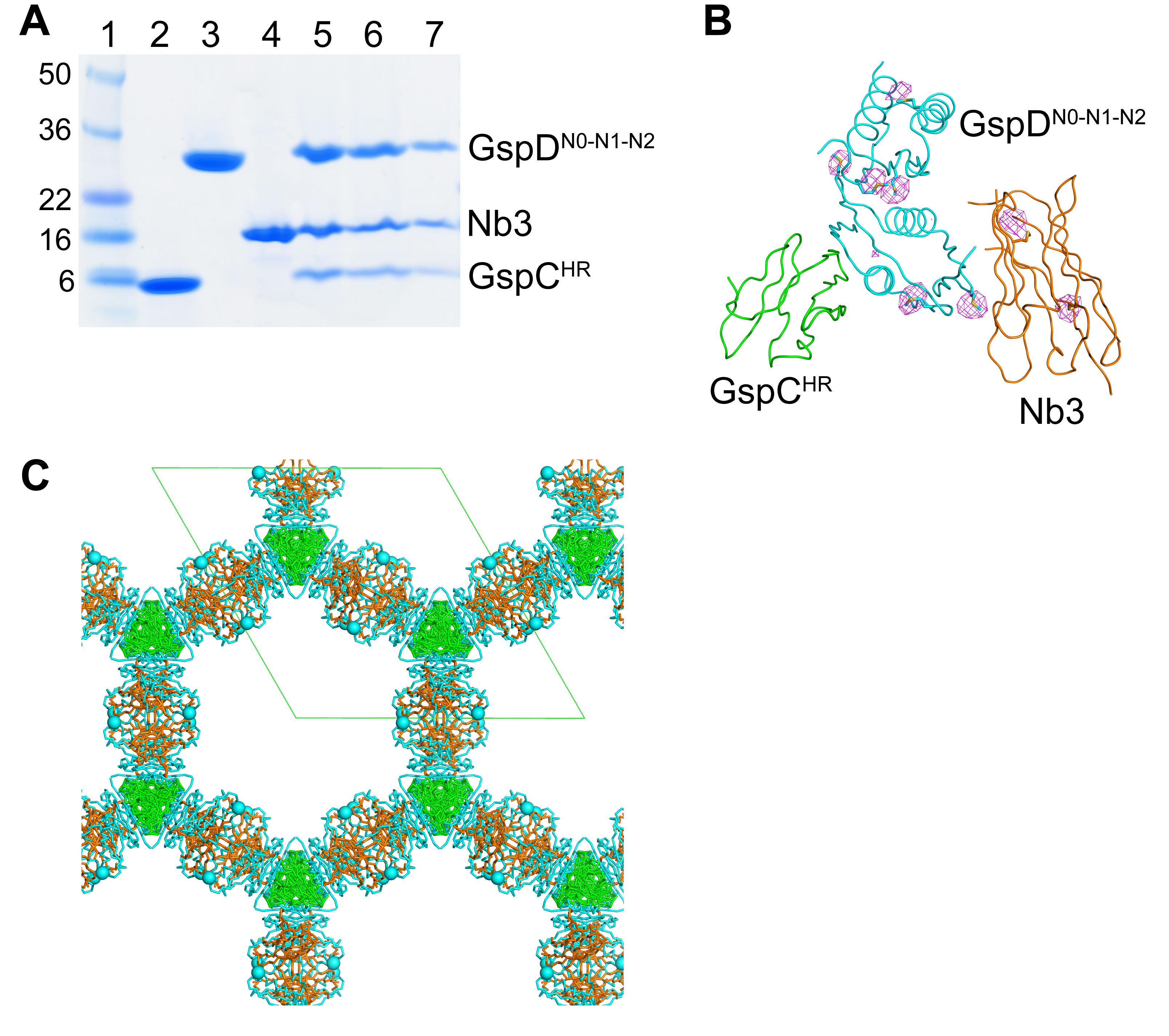

Supplement: Figure S2 — Crystal structure of the GspCHR–GspDN0-N1-N2–Nb3 ternary complex. (A) SDS-PAGE analysis of crystals. Lane 1, molecular weight standards; lane 2, purified GspCHR; lane 3, purified GspDN0-N1-N2; lane 4, purified Nb3; lane 5, GspCHR–GspDN0-N1-N2–Nb3 complex before crystallization; lane 6, drop which did not yield crystals; lane 7, recovered crystal after washing in artificial mother liquor. The GspDN0-N1-N2 chain is intact after crystallization. (B) Molecular replacement structure of the GspCHR–GspDN0-N1-N2–Nb3 complex. GspDN0-N1-N2 and Nb3 are Se-Met substituted proteins. Se-Met residues are shown as sticks. The anomalous difference map at the 3.5 σ level is shown as a magenta mesh and clearly indicates selenium sites. (C) Crystal packing of GspCHR–GspDN0-N1-N2–Nb3 viewed along the crystallographic c axis of space group P6122. GspCHR is in green, GspDN0-N1-N2 in cyan, Nb3 in orange. The Cα atoms of the last residue in the N1 domain (A165) are shown as cyan spheres. The N2 domains are facing long channels in the crystal lattice and are statistically disordered since SDS-PAGE analysis of dissolved crystals shows the full length of the GspDN0-N1-N2 chain [see lane 7 in (A) above]. (TIF) [file ppat.1002228.s002.tif]

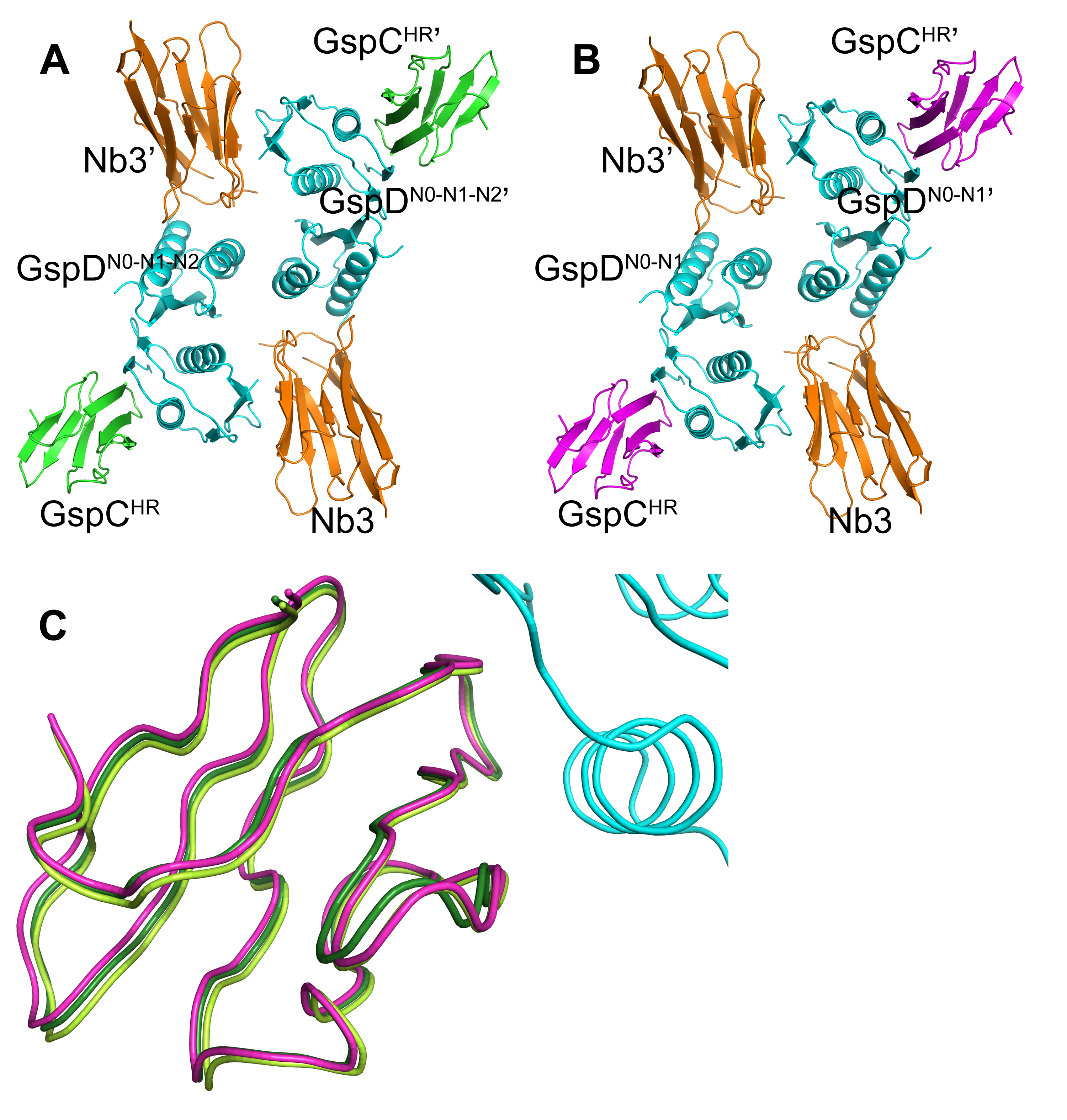

Supplement: Figure S3 — The GspCHR–GspDN0 interface in three crystal forms. The N0-N1 domains are colored cyan; the HR domains green and magenta; Nb3 nanobodies orange. (A) Two crystallographically related ternary GspCHR–GspDN0-N1-N2–Nb3 complexes in contact with each other in crystals with space group P6122. (B) Two crystallographically related ternary GspCHR–GspDN0-N1–Nb3 complexes in contact in crystals with space group P6422. Comparison with (A) above shows that the ternary complexes in these two crystal forms are very similar. The 2-fold crystallographic contacts are essentially the same in the two different crystal forms. (C) The GspCHR chain has the same orientation with respect to GspDN0 in three different crystal forms. The superposition of the three GspCHR–GspDN0 complexes is based on GspDN0-N1 only. The HR domain of GspCHR–GspDN0-N1 is depicted in dark green; the HR domain of GspCHR–GspDN0-N1-N2–Nb3 in light green; and the HR domain of GspCHR–GspDN0-N1–Nb3 in magenta. (TIF) [file ppat.1002228.s003.tif]

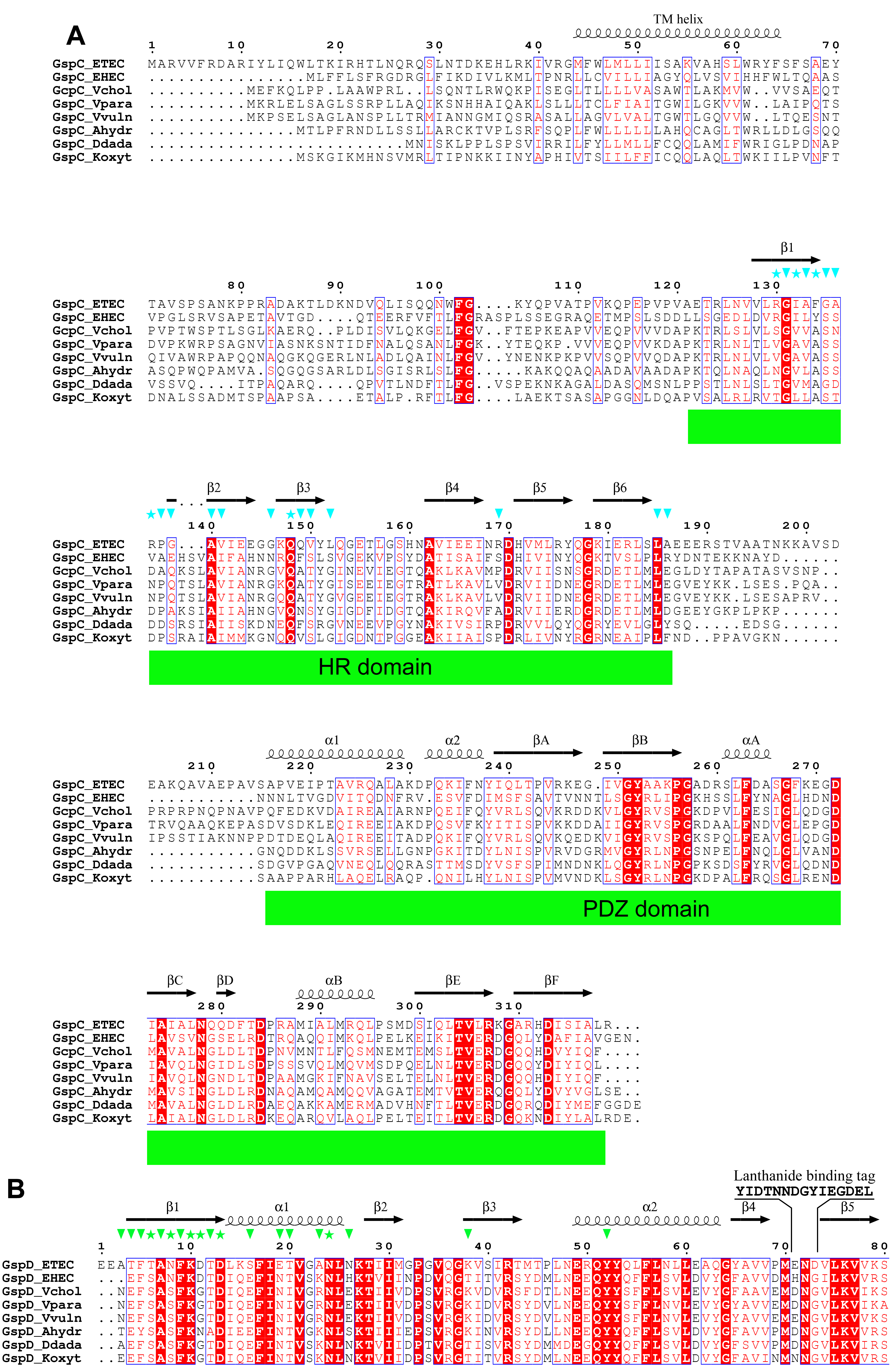

Supplement: Figure S4 — Sequence alignments of selected GspC proteins and GspDN0 domains. Residues that make intermolecular Van der Waals contacts and H-bonds in the ETEC GspCHR–GspDN0-N1 complex are labeled by triangles and stars, respectively. (A) Sequence alignment of GspC proteins. The secondary structure elements are shown at the top as determined from the ETEC GspCHR–GspDN0-N1 structure and the V. cholerae GspCPDZ structure (PDB 2I4S) [38]. (B) Sequence alignment of GspDN0 domains. The secondary structure elements are shown at the top as determined from the ETEC GspCHR–GspDN0-N1 structure. The position and sequence of the LBT are shown. (TIF) [file ppat.1002228.s004.tif]

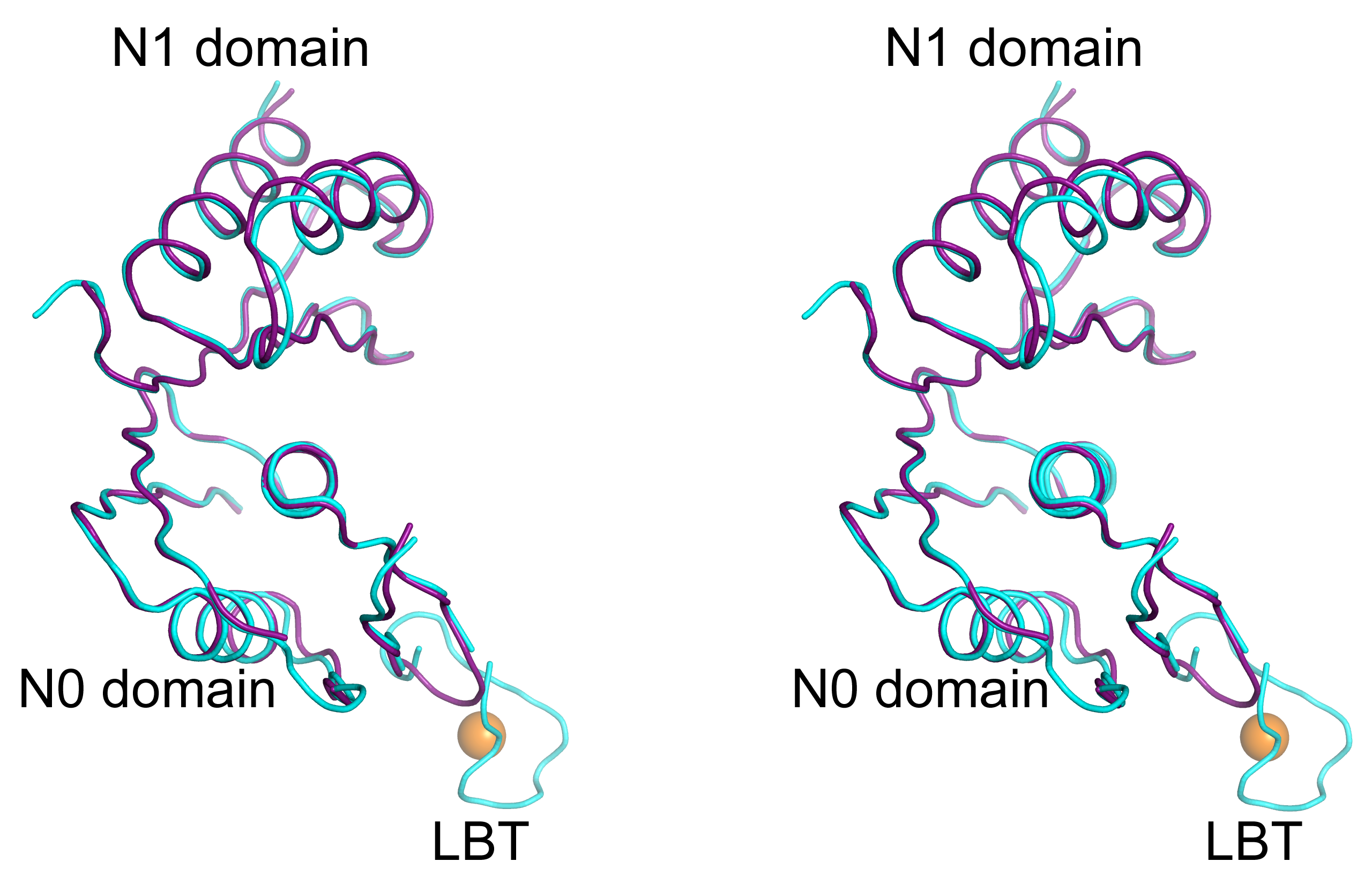

Supplement: Figure S5 — The structure of GspDN0-N1 is virtually the same in the GspCHR–GspDN0-N1 and GspDN0-N1-N2–Nb7 structures. A stereoview of a superposition of GspDN0-N1 from the GspCHR–GspDN0-N1 complex (cyan) and the GspDN0-N1-N2–Nb7 complex (purple, PDB 3EZJ) [24]. The superposition is based on the N0 domain only (r.m.s.d. 0.49 Å for 72 Cα atoms). The mutual orientation of the N0 and N1 domains is very similar indeed. (TIF) [file ppat.1002228.s005.tif]

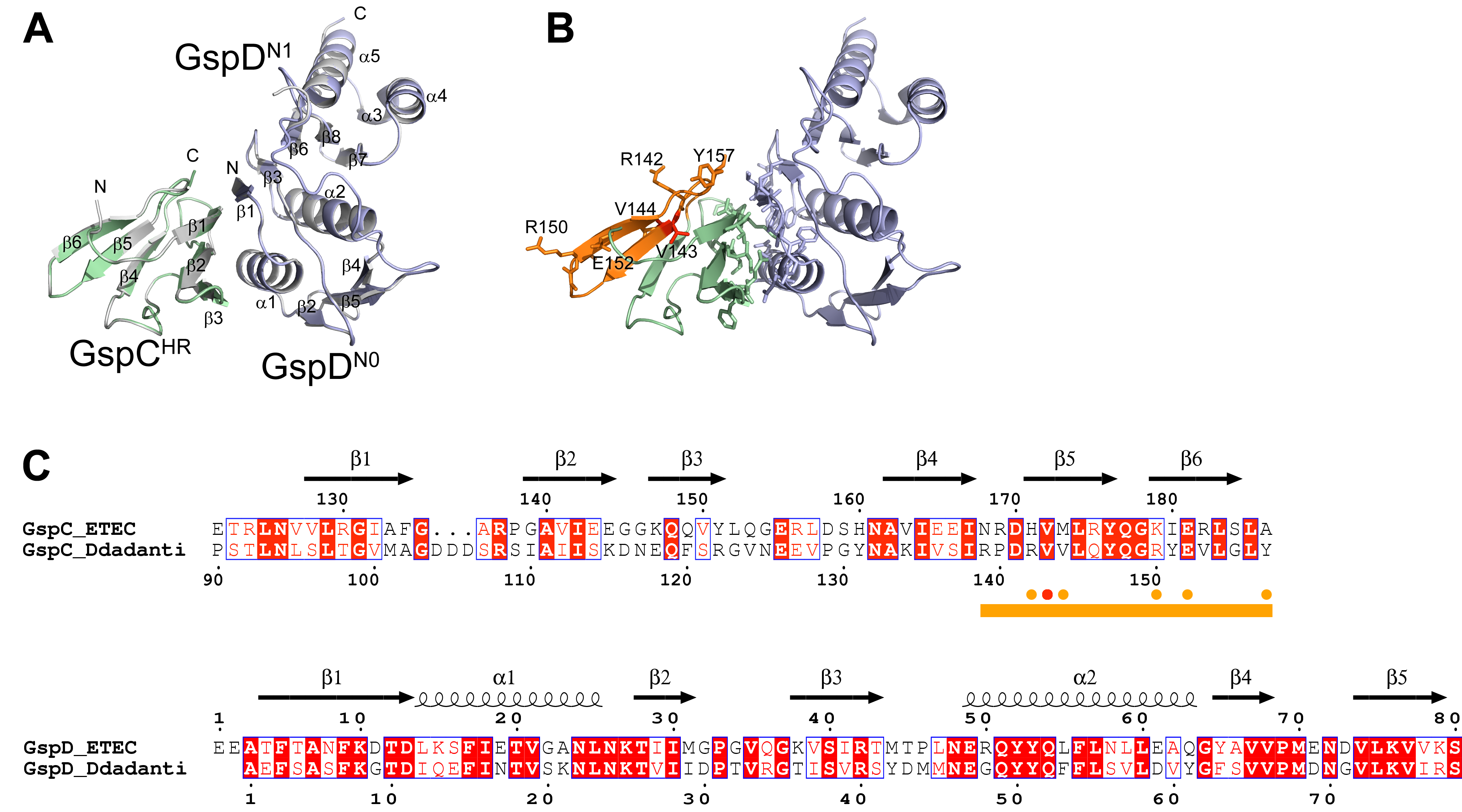

Supplement: Figure S6 — Analysis of the Dickeya dadantii GspCHR–GspDN0-N1 complex. (A) A homology model of the D. dadantii (previously Erwinia chrysanthemi) GspC–GspD complex. The structures of the HR domain of DdGspC (light green) and the N0-N1 domains of DdGspD (light blue) were obtained by homology modeling based on our new structure of the ETEC GspCHR–GspDN0-N1 complex (grey) as template, using the SWISS-MODEL server (http://swissmodel.expasy.org/) [79]. (B) Mutations of DdGspC. The residues in the interface of DdGspC-GspD in the homology model are shown as sticks. A previously suggested interaction region SIP (secretin interacting peptide) that corresponds to residues 139–159 is highlighted in orange [40]. The residues which have been subjected to mutational analysis (R142, V143, V144, R150, E152 and Y157) are shown as sticks and labeled. The mutant DdGspC proteins R142I, V144A, R150L, E152A and Y157A fully supported secretion of pectinases in D. dadantii. Note that, in contrast to the other residues, V143 is completely buried in the model and the substitution V143S leads to decreased secretion [40]. For further discussion see main text. (C) Sequence alignments of GspCHR and GspDN0 from ETEC and D. dadantii. Secondary structure elements are shown above alignment according to ETEC GspCHR–GspDN0-N1 crystal structure. A previously suggested interaction region SIP that corresponds to residues 139–159 is indicated by an orange bar. The residues which have been subjected to mutational analysis (R142, V143, V144, R150, E152 and Y157) are highlighted by circles. (TIF) [file ppat.1002228.s006.tif]
